# Effects of Different Transcranial Direct Current Stimulation Intensities over Dorsolateral Prefrontal Cortex on Brain Electrical Activity and Heart Rate Variability in Healthy and Fibromyalgia Women: A Randomized Crossover Trial

**DOI:** 10.3390/jcm13247526

**Published:** 2024-12-11

**Authors:** Mari Carmen Gomez-Alvaro, Narcis Gusi, Ricardo Cano-Plasencia, Juan Luis Leon-Llamas, Alvaro Murillo-Garcia, Maria Melo-Alonso, Santos Villafaina

**Affiliations:** 1 Grupo de Investigación en Actividad Física Calidad de Vida y Salud (AFYCAV), Facultad de Ciencias del Deporte, Universidad de Extremadura, Avenida de la Universidad s/n, 10003 Cáceres, Spain; maricarmengomezal@unex.es (M.C.G.-A.); leonllamas@unex.es (J.L.L.-L.); alvaromurillo@unex.es (A.M.-G.); svillafaina@unex.es (S.V.); 2 Instituto Universitario de Investigación e Innovación en Deporte (INIDE), Universidad de Extremadura, Av. de la Universidad s/n, 10003 Cáceres, Spain; 3 Neurofisiología Clínica, Hospital San Pedro de Alcántara, 10003 Cáceres, Spain; ricanopla@hotmail.com

**Keywords:** tDCS, chronic pain, fibromyalgia, EEG, HRV

## Abstract

People with fibromyalgia (FM) exhibit alterations in brain electrical activity and autonomic modulation compared to healthy individuals. **Objectives**: This study aimed to investigate transcranial direct current stimulation (tDCS) effects on brain electrocortical activity and heart rate variability (HRV), specifically targeting the dorsolateral prefrontal cortex in both healthy controls (HC) and FM groups, to identify potential differences in the responses between these groups, and to compare the effectiveness of two distinct tDCS intensities (1 mA and 2 mA) against a sham condition. **Methods**: Electroencephalography and electrocardiogram signals were recorded pre- and post-tDCS intervention. All participants underwent the three conditions (sham, 1 mA, and 2 mA) over three separate weeks, randomized in order. **Results**: No statistically significant baseline differences were found in the investigated HRV variables. In the FM group, 1 mA tDCS induced significant increases in LF, LF/HF, mean HR, SDNN, RMSSD, total power, SD1, SD2, and SampEn, and a decrease in HF, suggesting a shift toward sympathetic dominance. Additionally, 2 mA significantly increased SampEn compared to sham and 1 mA. In the HC group, sham increased DFA1 compared to 1 mA, and 2 mA induced smaller changes in SampEn relative to sham and 1 mA. No significant differences were found between FM and HC groups for any tDCS intensity. **Conclusions**: The effects of dlPFC-tDCS on HRV are intensity- and group-dependent, with the FM group exhibiting more pronounced changes at 1 mA and 2 mA. These findings emphasize the need for individualized stimulation protocols, given the variability in responses across groups and intensities.

## 1. Introduction

Fibromyalgia (FM) is a syndrome with a worldwide prevalence of 2.1%, with a higher incidence in women (4.3%) than in men (0.95%), in a global ratio 4:1 [1]. People with FM suffer from chronic, widespread, and persistent pain and other associated symptoms including stiffness, fatigue, sleep disorders, anxiety, depression, or mobility impairments [2]. Furthermore, people with FM tend to experience impaired cognitive functions such as memory, attention, processing speed, and executive functions [3,4,5], ultimately impacting their quality of life (QoL) [6].

In terms of brain electrical activity, electroencephalography (EEG) studies revealed altered resting-state brain electrical activity in patients with FM compared to individuals without the condition [7,8] and a greater brain deterioration when symptoms persist for years [9]. In fact, the electrical activity patterns of FM patients exhibit a distinct signature, allowing for discrimination between FM individuals and healthy controls (HC) based on detection-based EEG connectivity patterns. Significantly, in people with FM, the interconnections in the Delta and Beta frequency bands are remarkably limited, whereas the interconnections between the Alpha and Theta bands exclusively manifest in the frontotemporal regions. Furthermore, the functional interconnectivity between insular and frontal areas, such as the prefrontal cortex (PFC), measured by EEG, is greater in people with FM than in HC [10]. This difference in connectivity can be attributed to the variations in pain intensity observed between individuals with FM and controls [11].

Moreover, people with FM show altered autonomic modulation, with hyperactivation at rest and hypoactivation during stressful situations (dysautonomia) [12,13,14,15,16,17]. This autonomic modulation can be measured by heart rate variability (HRV) parameters that provide information about the balance between sympathetic and parasympathetic nervous system modulation [18]. In this regard, parasympathetic activity is reflected by high frequency power (HF), while sympathetic activity is linked to low frequency (LF) power and LF/HF ratio [19]. According to HRV parameters, research revealed that in healthy individuals, psychological stress triggers an elevation in heart rate (HR), a reduction in HF power, an increase in LF power, and an elevated LF/HF ratio [20]. Conversely, individuals with FM present low the root mean square of successive differences (RMSSD) and HF values and low parasympathetic activity at rest [21] and experience lower power levels in HF, LF, and total HRV parameters when confronted with mental stress. This could indicate a generalized decline in autonomic capacity that may be linked to impaired ascending pain inhibition originating from the cardiovascular system [17]. Consequently, individuals with FM encounter challenges in performing their daily activities [22]. In this context, the prefrontal cortex (PFC) is known as one of the brain regions associated with the modulation of autonomic nervous system (ANS) activity [23,24]. In this regard, significant correlations have been observed between HRV and the connectivity of specific brain regions, such as the amygdala, cingulate cortex, and PFC, suggesting that HRV is associated with more localized connectivity [25].

Therefore, it is crucial to consider the role of the PFC in FM, encompassing its involvement in brain electrical activity, brain interconnectivity, and HRV. Understanding the interplay between these factors is essential for comprehending the mechanisms underlying pain processing in individuals with FM. Accordingly, the identification of interventions capable of effectively modulating PFC activity becomes imperative in addressing this issue. One potential treatment option among the array of approaches available for FM management is transcranial direct current stimulation (tDCS) [26], which is a safe, painless, and effective non-invasive brain stimulation to neuromodulate cortical areas [27]. The effects of tDCS can be studied during stimulation (online effects) or following the stimulation session (offline) [28], and can last for an hour when more than 1 mA of intensity is applied for ten minutes or more [29]. This technique has been previously used in FM, mainly focused on the treatment of pain [30], being the cognitive effect less studied [31]. Nevertheless, it is important to consider the lack of studies investigating the impact of tDCS in PFC on cognitive function in FM, since this brain region has the capacity of elicit antinociceptive effects by means of inhibitory pain control pathways and enhancing cognitive and affective alterations due to its connection with the limbic system [31].

Previously, there have been some studies that applied tDCS in people with FM in PFC, specifically in the left dorsolateral prefrontal cortex (dlPFC), reducing pain and with no effect on sleep improvement [32,33,34] or QoL [35]. Regarding autonomic modulation, the application of tDCS on dLPFC has been previously reported as being able to modulate HRV parameters, HF and RMSSD, mostly in healthy individuals [36]. However, no studies have been found that have proven the efficacy of tDCS on HRV in people with FM. Meanwhile, both neuromodulation techniques, such as tDCS, and biofeedback, such as heart variability biofeedback (HRVB), reported to be able to alleviate FM symptoms. HRVB is a self-management technique within the domain of cardiorespiratory feedback training which works by decreasing the respiratory rate resulting in a slower heart rate, leading to longer intervals between heartbeats and improved HRV coherence. In this regard, it is important to highlight that the mechanism of action of HRVB on the nervous system is through stimulation of the peripheral nervous system, which alters central nervous system processing. In the case of tDCS, the effect is directly targeted to specific regions, and taking into account the role that dlFPC has in modulating the ANS, it could be a key region to stimulate by tDCS [19].

To our knowledge, there are no studies that focus on studying how brain electrical activity or certain parameters of HRV could be modified by using tDCS on PFC (dlPFC) in people with FM. Furthermore, no studies compared how different intensities may or may not produce different results within or between groups. In this respect, it is necessary to consider that the study of the intensity of tDCS is highly important in order to analyze its effects since there is still some controversy about the most appropriate intensity. It seems that applying higher intensities (e.g., 2 mA) is not always beneficial for anodal tDCS [37,38]. In healthy participants, few studies investigated these differences between intensities in PFC and have carried this out by applying tDCS during the task execution (online stimulation) in healthy participants [39,40]. Therefore, the present study aimed to explore the effects of different intensities of tDCS (sham, 1 mA, and 2 mA) on the dlPFC on brain electrical activity and HRV in people with FM and HC. Based on the scientific literature, we hypothesized that the montage on PFC (dlPFC) of tDCS would help to improve neurophysiological variables measured by EEG and HRV in women with FM and in HC. Moreover, there could be a different neurophysiological response in people with FM than in HC due to the differences in both brain electrical activity and autonomic modulation. Finally, we hypothesized that the results obtained are not directly related to the tDCS intensity applied.

## 2. Methods

### 2.1. Trial Desing

The present study was a double-blind, sham-controlled, cross-over design. All the conditions (sham, 1 mA, and 2 mA) were applied to all the participants in a randomized order. The order of application of the tDCS intensities (1 mA, 2 mA, and sham) was randomized using a random number generator (Random Number Generator tool; Google, LLC, Mountain View, CA, USA). There was a minimum of seven days between the conditions. A different intensity was applied each week. Participants completed the study in approximately 3 weeks.

All procedures were approved by the University of Extremadura research ethics committee (approval number: 192/2021) and were carried out in accordance with the Declaration of Helsinki [41]. The trial has been registered in the Protocol Registration and Results System (NCT05266989). A more detailed description of the research design is provided in the protocol, which is available online [42]. The study complies with the guidelines of the Consolidated Standards of Reporting Trials (CONSORT) [43].

### 2.2. Participants and Setting

Participants had to meet the following inclusion criteria in order to participate in the study: (a) be a female and aged between 30 and 75 years; (b) be able to communicate with the research staff; and (c) be diagnosed by a rheumatologist, according to the American College of Rheumatology’s criteria [2] (only for the FM group). The exclusion criteria were (a) suffered from a psychiatric disorder which can lead to cognitive impairment, a neurological disorder or brain injury; (b) were in pharmacological treatment for anxiety and depression; and (c) were pregnant.

In this regard, a total of 26 women (age: 48.50 (7.92)) participated in this study. Therefore, the total sample was collected during January/February 2022 and was divided into two groups: one group was composed by women with fibromyalgia (FM) (n = 13) that were recruited from the Association of Fibromyalgia (AFIBROEX) and the other group was composed by healthy women, known as healthy controls (HC) (n = 13). All intervention and assessments took place in the Faculty of Sport Sciences at the University of Extremadura (Caceres, Spain) between 9 and 13 a.m. The study was completed in March 2022. Participants gave written informed consent to participate in the study.

### 2.3. Intervention

The transcranial direct current stimulation (tDCS) was performed with an 8-channel hybrid EEG wireless neurostimulator (Starstim, NEuroelectrics, Barcelona, Spain). The sponge electrodes (Sponstim^®^, 5.65 cm diameter, 25 cm^2^ surface area) soaked in saline solution were used.

In order to stimulate the PFC, the anode was placed on the left dlPFC, corresponding to the F3 region of the international 10–20 electrode placement system. The return electrode was positioned on the contralateral supraorbital region, Fp2 [44,45,46,47,48,49,50,51,52]. Participants received anodal tDCS at an intensity of 1 mA or 2 mA with a ramp up and ramp down of 30 s each. A duration of 20 min was selected to ensure that the acute effects have at least one hour of duration [29]. For the sham condition, a double ramp of 30 s was used. This mode is included in the Neuroelectrics NIC1 program. These parameters for the sham condition were based on previous reports of perceived skin sensations, which generally disappear after 30 s [53,54].

### 2.4. Outcomes

#### 2.4.1. Primary Outcomes

EEG and electrocardiogram (ECG) signals for HRV were recorded using the Enobio^®^ device (Neuroelectrics, Cambridge, MA, USA) [55] and the Neuroelectrics^®^ instrument driver software (NIC version 1).

Drytrodes^®^ were used to obtain the EEG signal in 19 channels that were positioned according to the International System 10–20, in different scalp locations: frontal (Fz, Fp1, Fp2, F3, F4, F7, and F8), central (Cz, C3, and C4), temporal (T3, T4, T5, and T6), parietal (Pz, P3, and P4), and occipital (O1 and O2). Two electrodes placed in the earlobes were used as references. Impedance was kept below 10 KΩ during the songs. The EEG data were recorded at a 500 Hz sample rate. The EEGlab toolbox (MatLab) [56] was used to pre-process data. Data were downsampled to a 250 Hz sample rate. A 1 Hz high-pass filter was applied to remove the baseline drift. To reject bad channels and correct continuous data, the artifact subspace reconstruction (ASR) was used [57]. Then, bad channels were interpolated, and data were re-referenced to average. Adaptive mixture independent component analysis (AMICA) was used to perform an independent component analysis (ICA) [58]. Single equivalent current dipoles were estimated, and symmetrically constrained bilateral dipoles were searched. Independent components (ICs) whose dipoles’ residual variance was larger than 15% were removed, as well as those with dipoles located outside the brain. Once all sources of artifacts have been corrected, power spectral density was computed and banded into theta (4–7 Hz), alpha (8–12 Hz), and beta (13–30 Hz) frequency bands.

To collect the ECG signal, the Sticktrode^®^ electrode was placed in the V4-V5 position, in the fifth intercostal space, to identify RR intervals. The recording, in edf format, was exported to Kubios HRV software (v. 2.1) [59]. Different HRV variables was extracted: (a) time domain, including mean heart rate (mean HR), the standard deviation of a whole RR interval (SDDN), percentage of intervals >50 ms different from the previous interval (pNN50), and the square root of the mean of the squares of the successive differences of the interval RR (rMSSD); (b) frequency domain, which includes the ratio of low frequency (LF), the ratio of high frequency ratio (HF), the ratio of low frequency/the ratio of high frequency (LF/HF), and the sum of all spectra (total power); and (c) nonlinear measures such as detrended fluctuation analysis alpha 1 (DFA1), the dispersion and standard deviation of points perpendicular to the axis of line-of-identity in the Poincaré plot (SD1), the dispersion and standard deviation of points along the axis of line-of-identity in the Poincaré plot (SD2), and sample entropy (SampEn).

#### 2.4.2. Secondary Outcomes

The pain level was evaluated through a VAS for pain (0–100), asking by the intensity of pain referring to the day they were evaluated. In addition, participants were heighted and weighted using a stadiometer (SECA 225, SECA, Hamburg, Germany) in order to calculate the body mass index (BMI), and body composition was measured with a Tanita Body Composition Analyzer (TANITA BC-418MA, Tashkent, Uzbekistan). Age and medication intake were asked.

### 2.5. Procedure

For the collection of EEG and ECG signals, the following sequence was performed: (1) 5 min of baseline recording while participants were seated with open eyes and without specific breathing instructions; (2) the corresponding intensity for each day applied for 20 min (sham, 1 mA, and 2 mA); and (3) 5 min of EEG and ECG recording while participants were seated with open eyes and without specific breathing instructions.

### 2.6. Data Analysis

EEGlab study design was used to analyze the EEG signal comparing: (a) the pre tDCS and post tDCS for each intensity (sham, 1 mA, and 2 mA) within group (FM and HC) and (b) the effects of each intensity between groups. In addition, (c) the intensities post tDCS (sham, 1 mA, and 2 mA) within group and (d) the intensities post tDCS between groups were performed. Non-parametric analyses (permutation analysis) were computed with the false discovery rate correction (FDR) to control the type I error.

The Statistical Package for the Social Sciences (SPSS, version 24.0; IBM Corp., Armonk, NY, USA) was employed to analyze the effect of different tDCS intensities (sham, 1 mA, and 2 mA) in the HRV variables. Non-parametric analyses, Wilcoxon rank tests, were used to assess differences between the pre tDCS and post tDCS for each intensity (sham, 1 mA, and 2 mA) within the group (FM and HC). Furthermore, the Friedman test was performed to detect significant differences between tDCS intensities (sham, 1 mA, and 2 mA) effects and then a Wilcoxon signed-rank test was employed to conduct pairwise comparisons. Lastly, the difference between post and pre values was calculated for HRV variables. This difference was used to perform comparisons between groups in HRV at different intensities of applied tDCS by using the Mann–Whitney U test. The significance threshold (alpha = 0.05) was adjusted using the Benjamini–Hochberg method to manage the false discovery rate [60]. Effect sizes (r) were calculated, which are classified as follows: 0.5 is a large effect, 0.3 is a medium effect, and 0.1 is a small effect [61].

## 3. Results

### 3.1. Participant Characteristics

The main descriptive characteristics of the participants for FM and HC groups are shown in Table 1. The FM group reported a higher consumption of analgesics/relaxants, and more pain than the HC group, showing significant differences. Significant differences between groups were not observed in age, medication intake (diuretics, hypotensive, and other medication), or body mass index.

### 3.2. Electrocortical Brain Response to tDCS

**Figure 1:**
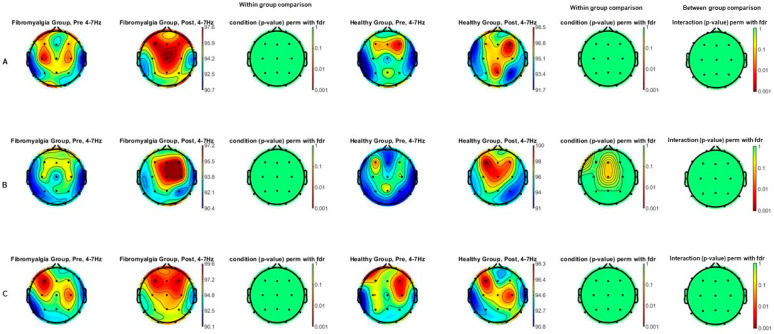
Theta power spectrum (4–7 Hz) topographic maps of the comparison of the effects of tDCS both between fibromyalgia and healthy groups and pre- post-tDCS intensities ((**A**): sham; (**B**): 1 mA; and (**C**): 2 mA) in each group separately. Statistically significant differences between pre- and post-tDCS intensities were not found in any of the healthy nor fibromyalgia groups independently. No differences in effects were observed between the fibromyalgia and healthy groups.

No statistically significant differences were observed in the analyses conducted at the baseline for the analyzed frequency bands. The theta power spectrum (4–7 Hz) comparisons topographic maps of the effects of each intensity of tDCS (A: sham; B: 1 mA; and C: 2 mA) between FM and HC groups (between group comparison) as well as comparisons between each intensity (A: sham; B: 1 mA; C: 2 mA), pre tDCS and post tDCS, for each group (within group comparisons) are shown in Figure 1. After applying the FDR correction for multiple comparisons, no significant differences were found (A, B, or C conditions) within or between groups.

**Figure 2:**
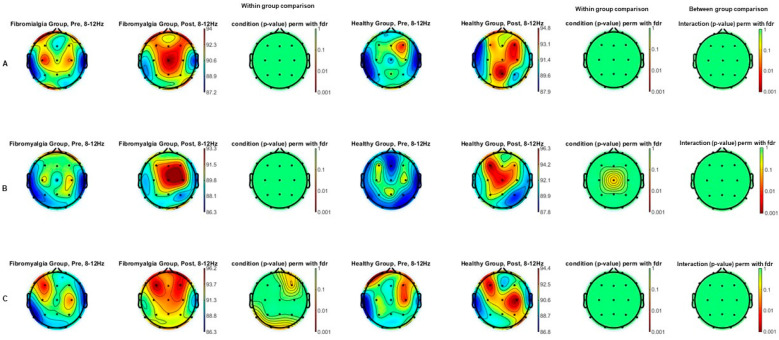
Alpha power spectrum (8–12 Hz) topographic maps of the comparison of the effects of tDCS both between fibromyalgia and healthy groups and pre- and post-tDCS intensities ((**A**): sham; (**B**): 1 mA; and (**C**): 2 mA) in each group separately. Statistically significant differences between pre- and post-tDCS intensities were not found in any of the healthy nor fibromyalgia groups independently. No differences in effects were observed between the fibromyalgia and healthy groups.

Figure 2 shows the alpha power spectrum (8–12 Hz) comparison topographic maps of the effects of each intensity of tDCS (A: sham; B: 1 mA; and C: 2 mA) between FM and HC groups (between group comparison) as well as comparisons between each intensity (A: sham; B: 1 mA; and C: 2 mA), pre tDCS and post tDCS, for each group (within group comparisons). After applying the FDR correction for multiple comparisons, no significant differences were found under any of the conditions (A, B, nor C) within and between groups. 

**Figure 3:**
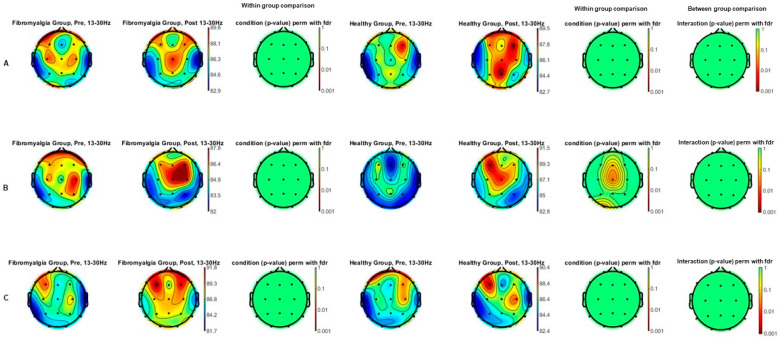
Beta power spectrum (13–30 Hz) topographic maps of the effects of tDCS both between fibromyalgia and healthy groups and pre- post-tDCS intensities ((**A**): sham; (**B**): 1 mA; and (**C**): 2 mA) in each group separately. Statistically significant differences in beta spectral power spectrum (*p* < 0.05) were found in Cz location under 1 mA condition (**B**) for the healthy group, with higher values after the tDCS protocol. Differences in effects between groups were not detected.

The beta power spectrum (13–30 Hz) comparison topographic maps of the effects of each intensity of tDCS (A: sham; B: 1 mA; and C: 2 mA) between FM and HC groups (between group comparison) as well as comparisons between each intensity (A: sham; B: 1 mA; and C: 2 mA), pre tDCS, and post tDCS, for each group (within group comparisons) are shown in Figure 3. After applying the FDR correction for multiple comparisons, statistically significant differences in beta spectral power spectrum (*p* < 0.05) were found in Cz location under B condition (1 mA) for the HC group, with higher values after tDCS protocol. Moreover, significant differences in effects between groups were not observed for any of the conditions.

**Figure 4:**
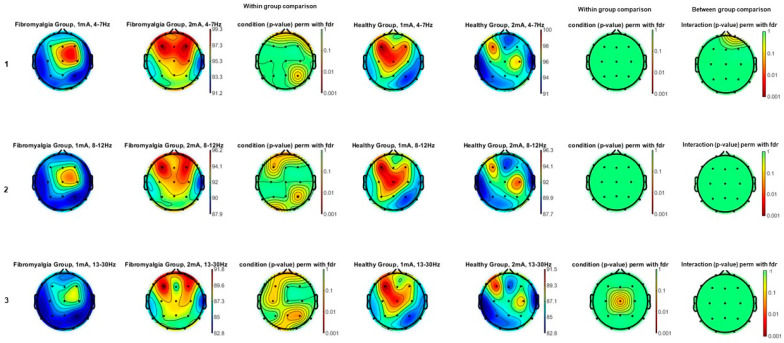
Theta (4–7 Hz)—(**1**), alpha (8–12 Hz)—(**2**), and y beta (13–30 Hz)—(**3**) power spectrum topographic maps of the comparison of the effects of tDCS between fibromyalgia and healthy groups and post-1 mA and 2 mA tDCS intensities. Statistically significant differences between the two intensities compared (1 mA and 2 mA) were found in the beta power spectrum in the healthy group for Cz location (*p* < 0.05). Differences in effects between groups were detected in theta power spectrum for Fp1 location (*p* < 0.05).

Figure 4 shows the theta (4–7 Hz; panel 1), alpha (8–12 Hz; panel 2), and beta (13–30 Hz; panel 3) power spectrum comparison topographic maps of the effects of tDCS between FM and HC groups (between group comparison) as well as comparisons between the post tDCS of two intensities (1 mA and 2 mA) for each group (within group comparisons). After applying the FDR correction for multiple comparisons, statistically significant differences between the two intensities compared (1 mA and 2 mA) were found in the beta power spectrum in the HC group for Cz location (*p* < 0.05), with higher values after the tDCS protocol. Furthermore, differences in effects between groups were detected in the theta power spectrum for Fp1 location (*p* < 0.05), with higher values after the tDCS protocol. 

### 3.3. HRV Response to tDCS

No statistically significant differences were found in the baseline HRV variables. Table 2 summarizes the comparisons between sham, 1 mA, and 2 mA tDCS in the FM and HC groups. The differences in stimulation effects between the FM and HC groups were also calculated for each variable to compare the impact of tDCS intensities.

In the FM group, the Wilcoxon signed-rank test showed that 1 mA stimulation significantly increased LF, LF/HF, mean HR, SDNN, RMSSD, total power, SD1, SD2, and SampEn, while HF decreased. In contrast, in the sham condition, only DFA1 showed statistically significant differences in the HC group.

The Friedman test in the HC group revealed significant differences between tDCS intensities for DFA1 and SampEn. Pairwise comparisons showed that DFA1 increased significantly with sham compared to 1 mA, while 2 mA produced smaller changes in SampEn compared to the increases observed with sham and 1 mA. In the FM group, significant differences between tDCS intensities were also found for SampEn (with LF and HF with a *p*-value = 0.05), with 2 mA inducing a greater increase compared to sham and 1 mA.

Lastly, the Mann–Whitney U test showed no significant differences between FM and HC groups for any tDCS intensity. However, prior to applying the Benjamini–Hochberg correction for multiple comparisons, a *p*-value below 0.05 was observed in response to 2 mA stimulation, specifically for SD2. This variable exhibited a medium effect size, with an increase observed in the FM group.

## 4. Discussion

The present study aimed to analyze the effects of tDCS on brain electrocortical activity and HRV, specifically targeting the dlPFC. Additionally, the study aimed to determine if there were differences in the response of HC and FM groups to this stimulation. The study also aimed to compare the efficacy of two distinct tDCS intensities (1 mA and 2 mA) in contrast to a sham condition.

In terms of brain electrocortical activity, EEG results show differences in beta spectral power, revealing heightened activation at Cz when tDCS was administered at 1 mA in HC. Furthermore, when comparing the effects of both intensities, discrepancies emerged within the same frequency band and location (Cz) for the same group. Specifically, in the HC group, 1 mA led to greater effectiveness compared to 2 mA. Moreover, when the effects of two intensities were compared between groups, significant differences were shown in the theta spectral power values in Fp1 between 1 mA and 2 mA, with higher levels in the FM group under 2 mA.

Regarding HRV variables, the HC group showed a significant difference when sham was applied. Additionally, when comparing the differences between intensities, 2 mA was observed to be more adept at reducing SampEn, although with minimal difference between 1 mA and 2 mA results in the context of SampEn. Furthermore, the increase in DFA1 was more pronounced with sham compared to 1 mA. However, in the FM group, both 1 mA and 2 mA appeared to exert positive effects on specific HRV variables, depending on the variable under examination. For instance, HF decreased with 1 mA and LF increased with 1 mA. Moreover, SDNN, RMSSD, PNN50, total power, SD1, SD2, and SampEn increased after 2 mA stimulation. Furthermore, the divergencies between the two intensities suggested that 2 mA generated the most substantial effects, inducing augmented SampEn in the FM group (with LF and HF power obtaining a *p*-value = 0.05). However, between FM and HC groups, differences were not found in the response to any intensity. However, after 2 mA, SD2 obtained a *p*-value lower than 0.05 before applying the Benjamini–Hochberg correction for multiple comparison, inhibiting a greater increase in the FM group. Thus, results may be taken with caution. To the best of our knowledge, this is the first study to analyze the EEG signal and HRV response prior to and post the application of tDCS (offline) targeting the dlPFC in people with FM compared to HC.

- Effects of Different Intensities on Brain Electrical Activity

In the context of brain electrical activity, our study revealed a distinct response to different tDCS intensities between the FM and HC groups. Therefore, the results are consistent with our hypothesis, as we expected a different response due to the differences observed in the resting brain electrical activity of people with FM with respect to HC [7,8,9]. However, these differences were not significant at baseline.

In HC, in our study, statistical differences were observed in beta spectral power, with a power spectrum in the central area augmented by applying 1 mA in HC. Beta frequency activity has been found to correlate with cognitive functioning during resting states [62]. In this regard, Song et al. [63] as well as Keeser et al. [64] previously reported an increase in beta activity in healthy individuals using tDCS. Firstly, Song, Shin, and Yun [63] observed that (online) tDCS over DLPFC contributes to the improvement in cognitive function by increasing beta frequency power, although with a different protocol than ours. Secondly, Keeser, Padberg, Reisinger, Pogarell, Kirsch, Palm, Karch, Möller, Nitsche, and Mulert [64] associated enhanced beta activity after (offline) tDCS with increased alertness and functional connectivity. In this case, the protocol was exactly the same as ours, except that the tDCS was administered at 2 mA only. Therefore, we can assert that our findings in HC are in line with others who observed an increase in beta activity, and that these results could potentially lead to an enhancement in cognitive performance. However, there is still some heterogeneity in the tDCS protocols for stimulating PFC. Moreover, it is important to mention that, in our study, the 1 mA intensity exhibited greater changes compared to the 2 mA intensity when comparing their respective effects. This observation may imply a heightened effectiveness of 1 mA over 2 mA in the HC for enhancing beta power. These findings agree with those reporting that higher intensity does not necessarily translate into better greater changes [65,66,67,68].

On the other hand, in the FM group, our study revealed significantly increased theta spectral power values in the frontal area when 2 mA tDCS was applied compared to the HC. Previously, it was observed that an increase in theta activity in the frontal area of the brain is associated with improved working memory [69,70]. Moreover, the theta band is considered a mechanism of cognitive control and focused attention [71,72], and its activity is directly linked to the PFC [73]. Individuals with FM exhibit heightened theta activity in frontal areas, which could contribute to persistent pain perception and may result from years of pain and fatigue [7,9]. Furthermore, chronic pain is associated with increased theta endogenous brain oscillatory rhythms at rest [74]. It has also been observed that after pain expressions, individuals with FM experience increases in theta and delta activity, which can affect inhibitory control and lead to poorer cognitive performance [75]. This phenomenon could be elucidated by the limited resourced theory, in which processing pain leads to alterations in the allocation and utilization of cognitive resources [76]. Additionally, in healthy individuals, the theta band is associated with increases in cognitive workload [77,78]. In this regard, in FM, González-Villar et al. [79] observed that the expected increase in theta during cognitive effort decreased in relation to the increase observed in healthy individuals. Moreover, Villafaina et al. [80] also observed that when comparing the performance in a single-task situation with a dual-task situation (performing two tasks simultaneously), the FM group showed no statistical differences in any frequency band, unlike the control group. The findings of this study led to the conclusion that individuals with FM fail to adjust their brain activity according to the task difficulty [80]. In line with this, González-Villar et al. [81] founded a reduction in theta and alpha power during a cognitive control task, indicating high levels of neural noise (random electrical fluctuations that impeded neural communication), thus confirming the presence of dyscognition in FM. When it comes to DLPFC-tDCS, Miller et al. [82] reported an increase in theta activity in frontal and left medial prefrontal brain areas after the stimulation, and Zaehle et al. [83] observed that anodal-tDCS during a working memory task led to an increment in oscillatory power in theta and alpha band. However, Gordon et al. [84] did not find any differences in resting-state EEG power spectrum in any of the tDCS conditions studied. It is important to clarify that all of these authors used DLPFC-tDCS setups different from ours and their studies were carried out with healthy individuals [82,83,84]. Taking all this information together, we can suggest that our results in the FM group are in line with those previously obtained by Miller, Berger and Sauseng [82] and Zaehle, Sandmann, Thorne, Jäncke, and Herrmann [83]. Therefore, an increase in theta activity in the frontal area could potentially lead to improved attention and cognitive enhancement, which may aid individuals with FM in mitigating symptoms of their condition.

- Effects of Different Intensities on HRV

The application of tDCS targeting the PFC has been previously investigated for its potential to modulate HR and HRV [36,85]. In this regard, Gu et al. [86] reported a decrease in HR and an increase in total power and LF y LF/HF ratio in healthy people using high definition tDCS (HD-tDCS) with F3 as the anode. The applied current intensity was set at 2 mA, and the stimulation duration was 20 min, differing partially from our experimental setup. In our study, the HC group exhibited significant reduction in the SampEn after 2 mA intensity. This intensity demonstrated greater changes than 1 mA in reducing mean HR, partially aligning with the findings of Gu, Chen, Lu, Dai, Hu, Xu, Geng, Zhu, Xu, Dai, and Shen [86]. However, diverse outcomes have been reported by other researchers in healthy populations. For instance, Nikolin et al. [87] showed an increase in HF during 15 min of 2 mA of bifrontal (F3, F4) tDCS and Karthikeyan et al. [88] observed that under anodal tDCS, both RMSSD and LF increased when applying the stimulation for 10 min with an intensity of 1 mA over F3 and Fp2 as the reference electrode. Both Nikolin, Boonstra, Loo and Martin [87] and Karthikeyan, Smoot, and Mehta [88] incorporated a working memory task in addition to tDCS. Moreover, other studies employing different stimulation montages in isolation have yield variable results. Vandermeeren et al. [89] applied 1 mA for 20 min with the anode at Fz and an extracephalic reference electrode on the right tibia and Brunoni et al. [90] used 1.5 mA, employing F3 as the anode and F4 as the cathode. Neither Vandermeeren, Jamart and Ossemann [89], nor Brunoni, Vanderhasselt, Boggio, Fregni, Dantas, Mill, Lotufo, and Benseñor [90] obtained specific tDCS effects on HRV parameters. As evidenced, substantial variability exists in the stimulation protocols targeting the DLPFC to induce HRV changes, contributing to challenges in attributing outcomes solely to the tDCS protocol used [91]. Furthermore, some researchers targeted the temporal cortex (TC), specifically T3 [92,93,94], aiming to stimulate the insular cortex, another region implicated in autonomic cardiac control [95,96]. In this regard, Petrocchi et al. [97] noted that the TC is positioned in closer proximity to the insular cortex than the DLPFC. This proximity could potentially elucidate the observed reduction in effects when stimulating the DLPFC in HC, aligning with our findings, as significant effects have been observed in only a limited subset of the parameters investigated. Interestingly, we have not found studies that measured DFA1 after tDCS. In our study, a noteworthy observation was the increase in DFA1 during sham stimulation. An increase in DFA1 reflects increased complexity and organization of HRV, indicating increased autonomic regulation [98]. The possibility of a placebo effect could be considered, as sham stimulation could have induced an autonomic response, increasing DFA1 and demonstrating a greater impact than 1 mA or 2 mA. Another possibility is that these results are due to non-specific responses or complex interactions [99].

People with FM reported decreased parasympathetic activity with reduced RMSSD, SDNN, PNN50, and HF and higher sympathetic activation with elevated LF and LF/HF [16,21,100]. Moreover, people suffering from chronic pain have been found to report a lower RMSSD [101] and some depressive symptoms are related to a decreased HRV [102]. To our knowledge, this is the first study to analyze the effects in autonomic modulation of tDCS in people with FM. Our findings demonstrate that both 1 mA and 2 mA intensities had significant effects on certain HRV variables, though the outcomes differed based on the applied intensity. Specifically, with 1 mA, LF and LF/HF increased, while HF decreased. Hence, the application of 1 mA tDCS would not necessarily have a positive effect, since people with FM have a lower HF, and a higher LF and LF/HF. Thus, in this scenario, the stimulation could potentially exacerbate these metrics. In line with this, Delaney and Brodie [20] suggested that under mental stress, healthy individuals would experience increased HR, LF, and LF/HF, and decreased HF. Reyes Del Paso, Garrido, Pulgar, Martín-Vázquez, and Duschek [17] also proposed that impaired ascending pain inhibition originating from the cardiovascular system, combined with reduced response to acute stress, could contribute to heightened pain sensitivity characteristic of FM. Therefore, our results could be linked to a possible mental stress response connected to pain circuits, potentially intensifying the autonomic stress prevalent in individuals with FM. Nevertheless, it is important to consider that interpretations within the classical framework of LF and HF measurements should be approached with caution [103]. Conversely, 2 mA stimulation resulted in significant increases in SDNN, RMSSD, and PNN50, contributing to an improvement in parasympathetic activity, which is typically compromised in FM. Furthermore, there were enhancements in total power, SD1, SD2, and SampEn values, collectively leading to improved HRV. Notably, when comparing the two intensities, the 2 mA stimulation exhibited more pronounced differences in comparison to the sham condition across SampEn. Notably, between FM and HC groups, differences were not found. However, prior to applying the Benjamini–Hochberg correction for multiple comparisons, a *p*-value below 0.05 was observed in response to 2 mA stimulation, specifically for SD2. This variable exhibited a medium effect size, with an increase observed in the FM group.

- Intra–Inter Individuality

According to our study, when comparing the same current intensity of tDCS between the two groups of study, different results are obtained. No differences were found in the analyses performed at baseline for the variables analyzed in both EEG and HRV variables. However, there were differences between groups indicating that women with FM take more analgesics/relaxants and have a higher level of pain than HC at baseline. In EEG results, 1 mA appears to be the most effective intensity in HC and 2 mA the most effective in FM. Moreover, to modify HRV parameters in FM, discernible effects are obtained with both 1 mA and 2 mA. Notably, the 2 mA intensity produced a moderate effect (according to SD2 results) with improvements in FM. However, few effects are observed in HC and through 2 mA. This is contrary to our hypothesis, which stated that the results obtained would not depend on the amount of current applied since the results obtained in FM depend on applying a higher intensity, in contrast to the HC.

The inconsistency in the results of studies comparing intensities has been studied in recent years, indicating that both inter- and intra-individual variability could be influencing the dose response of tDCS in a given population, highlighting the need for adjusted, individualized stimulation in order to help potentiate the effects of tDCS (76, 82, and 92–96). Related to tDCS, Esmaeilpour, Marangolo, Hampstead, Bestmann, Galletta, Knotkova, and Bikson [68] pointed out that for the same applied current, different effects may be produced in the brain of different individuals, hence the results would depend on the initial brain state of the subject. This is in line with the results of Splittgerber et al. [104], who reported that individual differences in cognitive performance and electrode montages influence effects of tDCS on neuropsychological performance. Moreover, the research of Tremblay et al. [105] showed that the variability in neurophysiological outcome seems to be related to the inter-individual variability in the neurophysiological response to tDCS. Therefore, as Chew et al. [106] reported in his study, due to this variability, it is important to interpret the results obtained with caution, especially when they are obtained through only one session per studied intensity.

To address the problem of inter-individual variability in tDCS studies, López-Alonso et al. [107] recommends increasing the sample size in studies which compare intensities. Other authors, such as Evans et al. [108], Kashyap et al. [109], and Van Hoornweder et al. [110], propose techniques that would help to target and apply individualized doses of tDCS to reduce this variability. It would be interesting to take into account these variability factors since we are analyzing two groups with different characteristics. By controlling for causes that may interfere with the results, it will help to have more consistent results. Moreover, considering the fluctuations in symptoms experienced by people with FM, systems that help to target and adjust doses to individual characteristics could be helpful in determining the most effective dose for each patient at the tDCS application time.

On the other hand, when it comes to modifying HRV parameters, it is important to consider the intra- and inter-individual variability that can influence such variables. The FM group would benefit from the application of both intensities, although the modified parameters would differ depending on the current. Limited results were obtained in HC when applying 2 mA. Firstly, concerning HRV, it is essential to consider how both intra- and inter-individual variability could influence stress response patterns. For instance, at an intra-individual level, the decision to focus solely on one axis of stress functionality, while recognizing the existence of multiple facets of functionality, may render the assessment insufficiently sensitive. This sensitivity could hinge upon the specific function being tested (in our case, only at rest). However, at an inter-individual level, biological factors (age, gender, and menstrual cycle phase), environmental factors (chronic stress), social factors, habits (smoking, drinking, sleeping, eating, and physical exercise), or physical factors could also be influencing these response patterns [111,112].

This study has some limitations. First, the sample is composed only of women, therefore, the results obtained could not be generalized to healthy men or men with FM. Moreover, sample size could be considered small for comparing effects of different intensities [107]. Likewise, only one session of each intensity was performed, which means that the results should be interpreted with caution, taking into account the intra–inter-individual variability mentioned in the discussion [106,111]. In addition, *p*-values were adjusted using the Benjamini–Hochberg correction to control for false positives in multiple comparisons. However, while this approach reduces the risk of type I errors, it may also increase the likelihood of type II errors, potentially masking some true positive findings.

## 5. Conclusions

Both HC and FM groups exhibited distinct responses in HRV to varying intensities of dlPFC-tDCS (sham, 1 mA, and 2 mA). In the FM group, 1 mA stimulation was associated with significant increases in LF, LF/HF, mean HR, SDNN, RMSSD, total power, SD1, SD2, and SampEn, along with a decrease in HF, indicating a potential shift toward sympathetic dominance. Conversely, 2 mA stimulation in the FM group led to a greater increase in SampEn compared to sham and 1 mA, suggesting differential effects of stimulation intensities on HRV complexity.

In the HC group, significant differences were observed in DFA1 and SampEn between tDCS intensities. Sham increased DFA1 compared to 1 mA, while 2 mA produced smaller variations in SampEn compared to the increases observed in sham and 1 mA. Importantly, no significant differences were detected between FM and HC groups for any tDCS intensity.

These findings suggest that the effects of dlPFC-tDCS on HRV are intensity- and group-dependent, with the FM group showing more pronounced changes at 1 mA and 2 mA. However, the variability observed across individuals and groups highlights the need for tailored stimulation protocols and further investigation to clarify the mechanisms underlying these responses.

## Figures and Tables

**Table 1:** Descriptive characteristics of the participants.

Measurements	FM Group Mean (SD)	HC Group Mean (SD)	*p*-Value	Effect Size
Sample size (N)	13	13		
Age (years)	49.92 (9.31)	47.08 (6.29)	0.572	0.111
Medication intake (%)				
Analgesics/Relaxants	8 (61.5%)	1 (7.7%)	0.005	0.555
Diuretics	1 (7.7%)	0 (0%)	0.317	0.196
Hypotensive	1 (7.7%)	1 (7.7%)	1.000	0.000
Others	9 (69.2%)	7 (53.8%)	0.429	0.155
BMI (kg/m^2^)	28.53 (7.39)	25.52 (3.96)	0.505	0.131
VAS for pain (0–100)	61.54 (20.65)	4.23 (11.11)	<0.001	0.844

Abbreviations: SD, standard deviation; FM, fibromyalgia; HC, healthy control; BMI, body mass index; and VAS: visual analogue scale.

**Table 2:** Effects of sham, 1 mA or 2 mA tDCS on HRV in HC and FM.

					Within Group Comparison				Between Group Comparison		
Variables	Group	Condition	Pre-tDCS Mean (SD)	Post-tDCS Mean (SD)	*p*-Value	Effect Size	Pairwise Comparisons		Condition	*p*-Value	Effect Size
SDNN (ms)	FM	Sham	29.83 (15.72)	30.20 (12.32)	0.530	0.174	0.779		Sham	0.913	0.021
		1 mA	25.99 (13.25)	29.4 (11.25)	0.142	0.500					
		2 mA	23.99 (9.62)	30.77 (12.77)	0.036	0.727			1 mA	0.939	0.025
	HC	Sham	26.82 (14.12)	28.99 (15.90)	0.676	0.359	0.926				
		1 mA	28.93 (19.08)	30.92 (22.33)	0.463	0.204			2 mA	0.520	0.297
		2 mA	26.48 (20.42)	28.89 (21.65)	0.221	0.339					
RMSSD	FM	Sham	27.96 (13.68)	28.43 (12.06)	0.530	0.174	0.205		Sham	0.913	0.053
		1 mA	24.50 (14.45)	26.02 (10.70)	0.362	0.305					
		2 mA	24.01 (11.57)	30.14 (14.88)	0.039	0.572			1 mA	0.914	0.146
	HC	Sham	28.40 (15.29)	29.06 (17.94)	0.807	0.068	0.116				
		1 mA	26.83 (19.44)	32.63 (29.95)	0.332	0.320			2 mA	0.980	0.065
		2 mA	27.25 (23.75)	31.58 (27.95)	0.150	0.514					
Mean HR	FM	Sham	72.93 (5.60)	72.00 (7.94)	0.530	0.239	0.558		Sham	0.913	0.064
		1 mA	76.51 (9.02)	73.72 (7.71)	0.142	0.566					
		2 mA	75.20 (5.33)	71.38 (8.30)	0.039	0.572			1 mA	0.939	0.015
	HC	Sham	72.54 (10.43)	71.78 (10.10)	0.676	0.184	0.050				
		1 mA	75.12 (11.89)	72.63 (12.34)	0.332	0.320			2 mA	0.980	0.005
		2 mA	75.70 (12.01)	71.63 (10.36)	0.150	0.494					
PNN50	FM	Sham	10.97 (14.05)	10.05 (12.23)	0.530	0.283	0.338		Sham	0.913	0.171
		1 mA	7.24 (14.75)	7.40 (10.15)	0.657	0.123					
		2 mA	6.16 (8.44)	13.77 (15.91)	0.038	0.653			1 mA	0.914	0.156
	HC	Sham	11.17 (13.63)	11.19 (14.96)	0.676	0.197	0.913				
		1 mA	8.80 (16.39)	12.45 (19.55)	0.332	0.370			2 mA	0.608	0.201
		2 mA	9.80 (18.55)	12.45 (20.14)	0.221	0.353					
LFnu	FM	Sham	55.03 (16.98)	50.94 (16.67)	0.875	0.109	0.050		Sham	0.212	0.317
		1 mA	49.05 (15.49)	58.28 (18.56)	0.045	0.631					
		2 mA	46.47 (20.90)	58.22 (13.24)	0.073	0.533			1 mA	0.141	0.327
	HC	Sham	42.46 (14.94)	53.10 (16.93)	0.111	0.533	0.232				
		1 mA	53.88 (20.72)	53.03 (18.49)	0.972	0.010			2 mA	0.172	0.337
		2 mA	44.71 (18.29)	40.49 (18.37)	0.861	0.107					
HFnu	FM	Sham	44.94 (16.98)	48.99 (16.69)	0.875	0.109	0.050		Sham	0.212	0.317
		1 mA	50.90 (15.49)	41.67 (18.58)	0.045	0.631					
		2 mA	53.48 (20.89)	41.73 (13.23)	0.073	0.533			1 mA	0.141	0.327
	HC	Sham	57.50 (14.94)	46.85 (16.93)	0.111	0.533	0.232				
		1 mA	46.05 (20.72)	46.92 (18.49)	0.972	0.010			2 mA	0.172	0.337
		2 mA	55.23 (18.26)	59.46 (18.37)	0.861	0.107					
LF/HF	FM	Sham	2.14 (3.25)	1.41 (1.27)	0.875	0.044	0.205		Sham	0.309	0.235
		1 mA	1.18 (0.78)	2.19 (2.44)	0.034	0.587					
		2 mA	1.40 (1.52)	1.61 (0.82)	0.249	0.320			1 mA	0.141	0.317
	HC	Sham	0.87 (0.57)	1.43 (0.94)	0.116	0.475	0.232				
		1 mA	1.95 (2.38)	1.43 (0.89)	0.972	0.029			2 mA	0.209	0.267
		2 mA	1.05 (0.84)	0.97 (1.061)	0.861	0.049					
Total power	FM	Sham	1181.87 (1210.30)	909.52 (759.08)	0.875	0.044	0.472		Sham	0.898	0.025
		1 mA	759.03 (836.85)	855.03 (541.24)	0.084	0.479					
		2 mA	620.17 (516.57)	1034.64 (733.41)	0.028	0.746			1 mA	0.489	0.136
	HC	Sham	873.21 (934.75)	981.75 (1150.39)	0.972	0.010	0.125				
		1 mA	1097.01 (1484.62)	1441.48 (2415.43)	0.348	0.475			2 mA	0.209	0.246
		2 mA	920.15 (1568.30)	1205.94 (2159.32)	0.184	0.552					
DFA1	FM	Sham	1.00 (0.22)	1.01 (0.18)	0.814	0.065	0.920		Sham	0.256	0.341
		1 mA	1.01 (0.16)	1.09 (0.23)	0.308	0.326					
		2 mA	0.96 (0.24)	1.02 (0.22)	0.173	0.378			1 mA	0.348	0.267
	HC	Sham	0.84 (0.20)	1.02 (0.23)	0.028	0.746	0.012	A > B 0.009			
		1 mA	1.00 (0.24)	0.98 (0.25)	0.552	0.165			2 mA	0.686	0.186
		2 mA	0.90 (0.23)	0.90 (0.29)	0.972	0.010					
SD1	FM	Sham	19.80 (9.69)	20.13 (8.54)	0.706	0.174	0.116		Sham	0.870	0.053
		1 mA	17.35 (10.24)	18.43 (7.58)	0.308	0.305					
		2 mA	17.00 (8.20)	21.34 (10.54)	0.039	0.572			1 mA	0.609	0.146
	HC	Sham	20.11 (10.83)	20.58 (12.71)	0.807	0.068	0.205				
		1 mA	19.00 (13.77)	23.11 (21.22)	0.498	0.320			2 mA	0.739	0.065
		2 mA	19.30 (16.82)	22.36 (19.78)	0.256	0.514					
SD2	FM	Sham	37.56 (15.51)	37.07 (20.36)	0.706	0.174	1.000		Sham	0.870	0.032
		1 mA	32.27 (15.91)	37.15 (14.47)	0.240	0.522					
		2 mA	29.17 (11.31)	37.68 (15.21)	0.018	0.727			1 mA	0.739	0.065
	HC	Sham	35.09 (19.33)	31.89 (17.36)	0.232	0.436	0.584				
		1 mA	36.02 (23.60)	36.77 (23.98)	0.552	0.204			2 mA	0.192	0.387
		2 mA	31.91 (23.78)	33.72 (24.03)	0.764	0.242					
SampEn	FM	Sham	1.60 (0.32)	1.72 (0.19)	0.468	0.435	<0.001	A > C <0.001 B > C <0.001	Sham	0.256	0.106
		1 mA	1.68 (0.13)	1.63 (0.16)	0.308	0.283					
		2 mA	1.59 (0.23)	1.78 (0.21)	0.018	0.727			1 mA	0.348	0.307
	HC	Sham	1.67 (0.14)	1.74 (0.25)	0.372	0.300	<0.001	A > C <0.001 B > C <0.001			
		1 mA	1.66 (0.27)	1.74 (0.20)	0.498	0.359			2 mA	0.739	0.085
		2 mA	1.73 (0.22)	1.72 (0.19)	0.866	0.126					

Note: A: sham; B: 1 mA; C: 2 mA. FM: fibromyalgia group; HC: healthy control group; SDNN = the standard deviation of all normal to normal RR intervals; RMSSD = the square root of the mean of the squares of the successive differences of the interval RR; HR = heart rate; PNN50 = percentage of intervals >50 ms different from the previous interval; HFnu = high frequency; LFnu = low frequency; LF/HF = low frequency (LF) ratio (ms^2^)/high frequency (HF) ratio (ms^2^); total power = the sum of all the spectra; DFA1: detrended fluctuation analysis alpha 1; SD1 = dispersion, standard deviation, of points perpendicular to the axis of line-of-identity in the Poincaré plot; SD2 = dispersion and standard deviation of points along the axis of line-of-identity in the Poincaré plot; and SampEn = sample entropy.

## Data Availability

The data of this research are confidential, since the participants signed in the informed consent form the confidentiality of their data.
